# Inhibition of IL-12 heterodimers impairs TLR9-mediated prevention of early mouse plasmacytoma cell growth

**DOI:** 10.3389/fmed.2022.1057252

**Published:** 2023-01-13

**Authors:** Mohamed F. Mandour, Pyone Pyone Soe, Anne-Sophie Castonguay, Jacques Van Snick, Jean-Paul Coutelier

**Affiliations:** ^1^Unit of Experimental Medicine, Université catholique de Louvain, Brussels, Belgium; ^2^Department of Clinical Pathology, Faculty of Medicine, Suez Canal University, Ismailia, Egypt; ^3^Department of Pathology, University of Medicine (1) Yangon, Yangon, Myanmar; ^4^Département de Pharmacologie et de Physiologie, Faculté de Médecine, Université de Montréal, Montréal, QC, Canada; ^5^Ludwig Institute, de Duve Institute, Université catholique de Louvain, Brussels, Belgium; ^6^de Duve Institute, Université catholique de Louvain, Woluwe-Saint-Lambert, Belgium

**Keywords:** cancer immunosurveillance, IL-12, plasmacytoma, NK/NKT cells, IFN-γ, TLR9, mesothelioma

## Abstract

**Introduction:**

Natural prevention of cancer development depends on an efficient immunosurveillance that may be modulated by environmental factors, including infections. Innate lymphoid cytotoxic cells have been shown to play a major role in this immunosurveillance. Interleukin-12 (IL-12) has been suggested to be a key factor in the activation of innate cytotoxic cells after infection, leading to the enhancement of cancer immunosurveillance.

**Methods:**

The aim of this work was to analyze in mouse experimental models by which mechanisms the interaction between infectious agent molecules and the early innate responses could enhance early inhibition of cancer growth and especially to assess the role of IL-12 by using novel antibodies specific for IL-12 heterodimers.

**Results:**

Ligation of toll-like receptor (TLR)9 by CpG-protected mice against plasmacytoma TEPC.1033.C2 cell early growth. This protection mediated by innate cytolytic cells was strictly dependent on IL-12 and partly on gamma-interferon. Moreover, the protective effect of CpG stimulation, and to a lesser extent of TLR3 and TLR7/8, and the role of IL-12 in this protection were confirmed in a model of early mesothelioma AB1 cell growth.

**Discussion:**

These results suggest that modulation of the mouse immune microenvironment by ligation of innate receptors deeply modifies the efficiency of cancer immunosurveillance through the secretion of IL-12, which may at least partly explain the inhibitory effect of previous infections on the prevalence of some cancers.

## 1. Introduction

Although a causal relationship between infectious agents and about two million cancers each year has been recognized (1), a few observations suggest also that some infections may decrease further cancer development through enhanced cancer immunosurveillance (2). Clinical surveys have reported an inverse relationship between history of infections and/or vaccinations and subsequent development of cancer (3, 4), including melanoma (5–7). Such an effect of infections may explain a lower incidence of some cancers, including multiple myeloma (8) in developing countries when compared to industrialized countries. Cancer development has been shown to be prevented by previous infection with lactate dehydrogenase-elevating virus (LDV), a virus inducing lifelong viremia, (9) and *Trypanosoma brucei* (10) in experimental mouse models of myeloma and mesothelioma (11). This led to the proposal that a particular form of “hygiene hypothesis” might apply to cancer development. Such a preventive effect of infections may, at least partly, explain the lower incidence of some cancers in developing countries where infection incidences are high, when compared to industrialized countries.

The efficiency of cancer immunosurveillance has been shown to depend on the activation of the immune system, and especially on the secretion of gamma-interferon (IFN-γ) by innate lymphoid cytotoxic cells such as natural killer (NK) cells and NK/T cells (12, 13). CD8+ cells have also been shown to provide innate IFN-γ production when stimulated by the appropriate cytokines (14) and could therefore also be involved in cancer immunosurveillance. IFN-γ has indeed been shown to be at least partly responsible for the preventive effect of LDV and *T. brucei* on further mouse plasmacytoma development (9, 10). It may therefore be postulated that the effect of infections on cancer immunosurveillance correlates with their ability to modulate the immune microenvironment of their host, and especially the capacity of NK cells to destroy tumor cells.

Since infections have complex effects on their host immune system, it is difficult to assign to a single mechanism their modulation of cancer development. However, the first interactions between invading infectious agents and the immune system involve recognition of pathogen-associated molecular patterns (PAMPs) by pattern recognition receptors (PRRs). Among these PRRs, toll-like receptors (TLRs) have been reported to recognize PAMPs from bacteria, viruses, parasites, and fungi and to initiate potent early immune signals (15). These early signals induced by the interactions between PAMPs and TLRs include the secretion of cytokines, and especially of interleukin-12 (IL-12) that, in turn, will trigger the activation of innate lymphoid cells. Since activation of NK/Natural-killer T cells (NKT) cells through ligation of TLRs, and especially TLR7, 8 and 9 that recognize infectious agent genetic material in endosomes, has been shown to enhance anti-tumor responses (16), we investigated the potential of these TLRs in enhanced cancer immunosurveillance. Our results indicate that activation of TLRs, and especially CpG stimulation increase early prevention of plasmacytoma and mesothelioma growth. This effect was strongly dependent on the secretion of IL-12.

## 2. Materials and methods

### 2.1. Animals

Specific pathogen-free BALB/c female mice were bred at the Ludwig Institute for Cancer Research or were obtained from Janvier Labs and used when 7–10 weeks old. The total number of 715 mice was used for this project. This mouse strain was chosen since TEPC.1033.C2 and AB1 cells were derived from BALB/c animals. All experimental protocols and animal handling were approved by the local commission for animalcare: Comité d’Ethique facultaire pour l’Expérimentation Animale–Secteur des Sciences de la Santé–Université catholique de Louvain (ref. 2014/UCL/MD/008 and 2018/UCL/MD/007).

### 2.2. Tumor cells

Plasmacytoma TEPC.1033.C2 cells, originally obtained from Dr M. Potter (17) were cultured in Iscove’s Modified Dulbecco’s Medium (IMDM) (Gibco, Life technologies, Grand Isle, NY, USA) with 10% fetal bovine serum (FBS, Gibco, Life technologies, Grand Isle, NY, USA), 50 U/ml penicillin G and 50 μg/ml streptomycin (Gibco, Life technologies). Cells were collected by brief trypsinization, washed twice with phosphate buffered saline (PBS) and injected i.p. at a dose of 3–4 × 10^4^ living cells (counted using trypan blue staining) in 500 μl PBS.

AB1, a mouse mesothelioma cell line derived from mouse lung (18) was obtained from Sigma-Aldrich (Public Health England, general cell collection, Ref. 10092305) and maintained in RPMI 1,640 medium containing 25 mM HEPES, 5% FBS, 50 U/ml penicillin G, 50 μg/ml streptomycin, and 2 mM L-Glutamine (Gibco, Life technologies). Exponentially growing cells were collected by brief trypsinization, washed twice with PBS and injected i.p. at a dose of 0.5 × 10^6^ living cells, counted using trypan blue staining, in 500 μl PBS.

### 2.3. TLR ligands

Mice were injected i.p. with TLR2, 3, 4, 7/8 and 9 ligands for two successive days before inoculation of tumor cells. Peptidoglycan from *Methanobacterium* sp. (Sigma-Aldrich, ref. 78721) was injected at a dose of 10 μg/mouse. Polyinosinic–polycytidylic acid sodium salt [Poly (I:C) (Sigma-Aldrich, ref. P1530)] was injected at a dose of 50 μg/mouse. Lipopolysaccharide (LPS) from *Escherichia coli* 0111:B4 purified by phenol extraction (Sigma-Aldrich, ref L2630) was injected at a dose of 10 μg/mouse. R848 (resiquimod) (Enzo, ref. ALX-420-038-M005) was injected at a dose of 50 μg/mouse. CpG-C DNA (ODN 2395) (Hycult biotech, ref HC4041) was injected at a dose of 10 μg/mouse.

### 2.4. Antibodies, NK cell depletion, and cytokine neutralization

Anti-asialoganglioside-GM1 (anti-ASGM1) polyclonal antibody from immunized rabbit was prepared and used following a protocol shown previously to successfully deplete NK cells and to suppress their function (19). *In vivo* NK cell depletion was achieved by i.p. injection of 2 mg anti-ASGM1 in 500 μl saline 2 days before tumor cell administration, followed by injection of 1 mg anti-ASGM1 in 300 μl saline the day of tumor inoculation.

F3 rat anti-mouse IFN-γ monoclonal antibody (mAb) (20, 21), purified with protein G-sepharose beads, was injected i.p. into mice at a dose of 500 μg 1 day before and 6 days after TLR9 ligation. MM12A1.6 mouse IgG2a anti-IL-12 mAb (22, 23) was injected i.p. into mice at a dose of 500 μg 1 day before and 6 days after TLR9 ligation. C1407C3 mouse IgG2a control mAb was injected at the same times and doses.

### 2.5. Isolation of spleen and peritoneal cells

After mice euthanasia, spleen was transferred to be processed into a sterile 35 mm petri dish containing 5 ml of sterile dissection medium (PBS + 1 mM EDTA). Then, spleen was mechanically crushed using the flat end of a sterile 3 cc syringe plunger by gentle circular motions. The released splenocytes were strained and filtered through a 70 μm cell strainer on a sterile 15 ml conical tube. After being centrifuged for 10 min at 8°C, (1,200 rpm), red blood cells were completely lysed using 2.5 ml Ammonium-Chloride-Potassium (ACK) lysis buffer [0.15 M NH_4_Cl (Merck, #1.01149), 10 mM KHCO_3_ (Merck, #1.00119), 0.1 mM Na_2_EDTA (Sigma, #E5134), (pH 7.3)] for 5 min on ice. The tube was toped up with 5 ml PBS, centrifuged at 1,200 rpm for 10 min. After discarding the supernatant, the cell pellet was washed 1–2 times and resuspended in 1 ml PBS. Cells were counted and assessed for viability using trypan blue stain.

Peritoneal cells were harvested by washing the peritoneal cavity with 2 × 5 ml ice-cold PBS supplemented with 5% fetal bovine serum (FBS, Gibco, Life Technologies, Grand Isle, NY, USA), 50 U/ml penicillin G, 50 μg/ml streptomycin (Gibco, Life Technologies), and 2 mM EDTA (Sigma-Aldrich, St. Louis, MO, USA). The cells were washed, resuspended in Iscove’s Modified Dulbecco’s Medium (IMDM, Gibco Life Technologies) supplemented with 10% FBS, non-essential amino acids, 50 U/ml penicillin G, and 50 μg/ml streptomycin. Cells were counted and assessed for viability using trypan blue dye before further processing and staining.

### 2.6. Flow cytometry

Flow cytometry analysis of NK cells and IFN-γ -producing cells was carried out using BD-FACSVerse machine (Becton Dickinson, Franklin Lakes, NJ, USA). Peritoneal cells were first incubated for 4 h at 37°C with 10 μg/ml monensin (Biolegend, San Diego, CA, USA; Cat# 420701). γ-block was done using purified anti-mouse CD16/32 antibody (2.4G2; Biolegend, Cat# 101301). NK cells were labeled by surface staining with 1.0 μg APC-labeled anti-mouse CD49b mAb (DX5; Biolegend, Cat# 108909) per 10^6^ cells. Anti-CD49b mAb was used, since it recognizes NK and NKT BALB/c cells. For intracellular labeling of IFN-γ, cells were fixed and permeabilized using Cyto-Fast™ Fix/Perm Buffer Set 111 (Biolegend, Cat# 426803) followed by staining with PE-labeled anti-IFN-γ mAb (XMG1.2; Biolegend, Cat# 505807). Data were analyzed by using FlowJo Software 9.8.1 (Tree Star, Ashland, OR, USA).

### 2.7. Statistical analysis

Results are expressed as means ± standard error of mean (SEM). When appropriate, one-way or two-way ANOVA tests with Bonferroni correction were performed using Prism 6 software (GraphPad Prism, La Jolla, CA, USA). Survival curves were analyzed using Log-rank (Mantel–Cox) test.

## 3. Results

### 3.1. Prevention of plasmacytoma early development after TLR ligation

**FIGURE 1:**
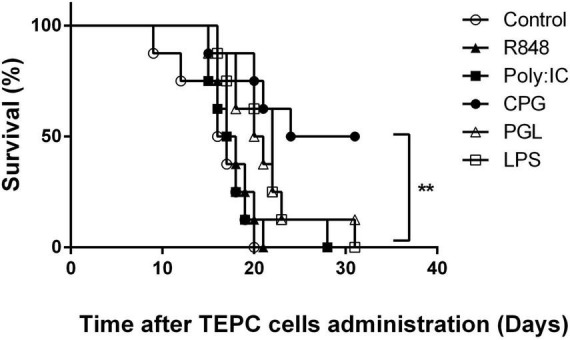
Effect of toll-like receptor (TLR) ligand administration on early plasmacytoma growth. Survival of groups of BALB/c mice (*n* = 8) either mock-treated (open circles) or treated with Poly (I:C) (closed squares), R848 (closed triangles), CpG-ODN (closed circles), lipopolysaccharide (LPS) (open squares), and peptidoglycan (open triangles) for two successive days before tumor administration. Groups were monitored daily after i.p. administration of plasmacytoma TEPC.1033.C2 cells. Experiment representative of three independent experiments. **P = 0.0013 by Log-rank (Mantel–Cox) test.

Previous observations indicated that infections, including with a virus such as LDV occurring prior to tumor cell administration prevented early development of plasmacytoma, a mouse model of multiple myeloma (9). Similarly, decrease of cancer incidence has been reported in humans after various infectious stimuli (3–8). To extend in an animal model these observations to conditions mimicking the effect of diverse infections on the immune system, we treated mice with TLR ligands before administration of cancer cells. After administration of TEPC.1033.C2 plasmacytoma cells, all control mice died within 20 days (Figure 1). In contrast, mice treated with TLR9 ligand CpG-ODN were protected against plasmacytoma development and half of them were still alive after 1 month without clinical signs of cancer development (Figure 1, *p* = 0.0013). A similar preventive effect of CpG-ODN was obtained in three independent experiments. Despite a statistically significant difference between survival of control mice and those treated with bacterial peptidoglycan and LPS, TLR2 and TLR4 ligands, (Figure 1, *p* = 0.01 and 0.004, respectively), the preventive effect of these molecules was much lower than after CpG-ODN treatment. In addition, TLR3 ligand Poly (I:C) and TLR7/8 ligand R848 had no preventive effect against plasmacytoma growth (Figure 1, *p* = 0.56 and 0.28, respectively). Since CpG-ODN stimulation induced the most efficient prevention of plasmacytoma early growth, we focused our analysis on this treatment.

### 3.2. Involvement of innate lymphoid cytotoxic cells in TLR9-mediated prevention of early plasmacytoma growth

**FIGURE 2:**
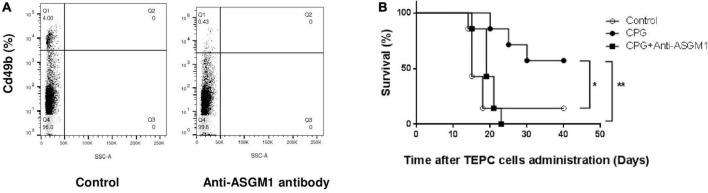
Role of innate lymphoid cytotoxic cells in toll-like receptor (TLR)9-mediated protection against plasmacytoma growth. **(A)** Flow cytometry analysis of CD49b+ cells in pooled spleen cells from 2 BALB/c mice with and without treatment with 2 mg anti-ASGM1 antibody. Experiment representative of two independent experiments. **(B)** Survival of groups of BALB/c mice (*n* = 7) either mocked-treated (open circles) or treated only with CpG-ODN (closed circles), or with CpG-ODN and anti-ASGM1 treatment (closed squares), was monitored daily after i.p. administration of TEPC.1033.C2 cells. Experiment representative of three independent experiments. *P = 0.0153 and **P = 0.0015 by Log-rank (Mantel–Cox) test.

Innate cytotoxic cells have been reported to play a major role in cancer immunosurveillance (13) and prevention of early cancer growth after LDV infection relies on these cells (9, 19). To determine whether these cells were also involved in TLR9-mediated plasmacytoma growth prevention, we treated mice with a polyclonal anti-ASGM1 antibody. As shown in Figure 2A, this treatment effectively suppressed spleen CD49b+ cells (4% CD49b+ cells without anti-ASGM1 treatment versus 0.43% CD49b+ cells after treatment). Anti-ASGM1 antibody administration resulted in almost complete abolition of CpG-ODN-mediated protective effect on plasmacytoma development after TEPC.1033.C2 cell administration (shown in Figure 2B for one representative experiment among three; difference with and without treatment: *p* = 0.0015). Therefore, innate cytotoxic lymphoid cells were required for protection against early plasmacytoma growth induced by CpG stimulation.

### 3.3. Role of IFN-γ and IL-12 in TLR9-mediated prevention of early plasmacytoma growth

**FIGURE 3:**
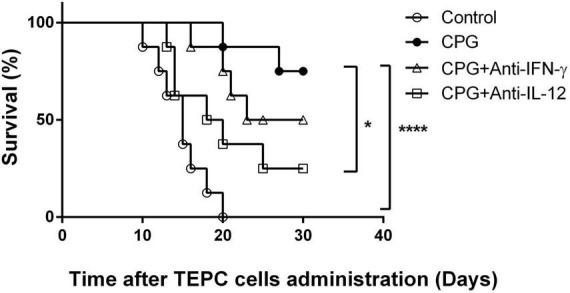
Role of IFN-γ and interleukin-12 (IL-12) in toll-like receptor (TLR)9-mediated protection against early plasmacytoma growth. Survival of groups of BALB/c mice (*n* = 8) either mocked treated (open circles) or CpG-ODN treated, without (closed circles) or with anti-IFN-γ (open triangles) and anti-IL-12 (open squares) treatment, was monitored daily after i.p. administration of TEPC.1033.C2 cells. Experiment representative of two independent experiments. *P = 0.023 (CpG vs. CpG+ anti-IL-12) and *P* = 0.24 (CpG vs. CpG+ anti-IFN-γ) by Log-rank (Mantel–Cox) test. ****P≤0.0001.

It has been reported that CpG stimulation in mice induces both IL-12 and IFN-γ production (24). Moreover, those cytokines were involved in LDV-induced prevention of plasmacytoma growth (9). We therefore analyzed the role of these cytokines in TLR9-mediated prevention of early plasmacytoma development by treating mice with the neutralizing F3 anti-IFN-γ mAb and a specific anti-IL-12 mAb recognizing the heterodimeric cytokine (MM12A1.6) (23). After CpG-ODN administration, IL-12 neutralization resulted in a significant decrease in the survival after plasmacytoma inoculation (Figure 3, *p* < 0.023). The suppression of CpG-ODN induced plasmacytoma growth prevention after IFN-γ neutralization was less important and did not reach significance (Figure 3, *p* = 0.61). These results are representative of two independent experiments.

### 3.4. Effect of CpG-ODN stimulation on prevention of early mesothelioma growth

**FIGURE 4:**
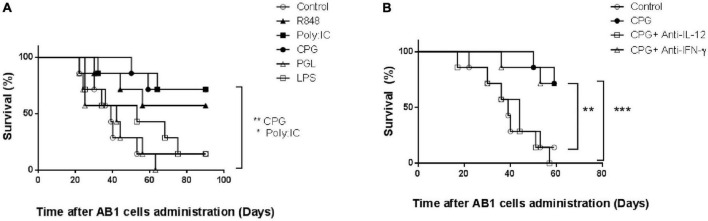
Effect of toll-like receptor (TLR)9 ligation and interleukin-12 (IL-12) secretion on protection against early mesothelioma growth. **(A)** Survival of groups of BALB/c mice (*n* = 8) either mock-treated (open circles) or treated with Poly (I:C) (closed squares), R848 (closed triangles), CpG-ODN (closed circles), lipopolysaccharide (LPS) (open squares) and peptidoglycan (open triangles) for two successive days before tumor administration. Groups were monitored daily after i.p. administration of mesothelioma AB1 cells. Experiment representative of three independent experiments. **P = 0.0096 (CpG vs. control), *P = 0.02 [Poly (I:C) vs. control], respectively, by Log-rank (Mantel–Cox) test. **(B)** Survival of groups of BALB/c mice (*n* = 7) either mocked treated (open circles) or treated for two successive days with CpG-ODN without (closed circles) or with anti-IFN-γ (open triangles) or anti-IL-12 (open squares) treatment, was monitored daily after i.p. administration of AB1 cells. Experiment representative of two independent experiments. ***P = 0.0008 (CpG vs. CpG+anti-IL-12), **P = 0.0096 (CpG vs. control), and *P* = 0.023 (CpG+anti-IFN-γ vs. control), respectively by Log-rank (Mantel–Cox) test.

Lactate dehydrogenase-elevating virus infection has been shown to protect against mesothelioma early growth through mechanisms similar to those involved in protection against plasmacytoma (11). To determine whether our observation of protection against early plasmacytoma growth after CpG-ODN stimulation could be extended to other tumors, we repeated therefore our experiments after administration of AB1 mesothelioma cells. A similar preventive effect against tumor development was observed in mice treated with CpG-ODN (Figure 4A, *p* = 0.0096). Administration of Poly (I:C) and of R848 prevented also mesothelioma development (Figure 4A). This preventive effect of TLR3, 7/8 and 9 ligands was observed in three independent experiments. In contrast, neither TLR4 nor TLR2 stimulation with LPS and bacterial peptidoglycan, respectively, could prevent mesothelioma development (Figure 4A, *p* = 0.62 and 0.91, respectively).

The role of IL-12 and IFN-γ in this TLR9-induced protection against mesothelioma early growth was also analyzed with the same neutralizing antibodies. As shown in Figure 4B, IL-12 neutralization resulted in a significant decrease in the protective effect of CpG-ODN stimulation (*p* = 0.0008). In contrast, IFN-γ neutralization did not modify TLR9-mediated prevention of mesothelioma growth (*p* = 0.93). Interestingly, neutralization of both IFN-γ and IL-12 suppressed completely prevention of mesothelioma growth after R848 treatment and largely after Poly (I:C) treatment (data not shown).

## 4. Discussion

In addition to their causative role in tumor development, infections have been reported to prevent further development of some cancers (3–7), a phenomenon that has been compared to the “hygiene hypothesis” or “old friends hypothesis” (2, 25), which could explain the increasing incidence of allergies and autoimmune diseases in industrialized countries with reduced prevalence of many infections. Such a cancer-related “hygiene hypothesis” could partly explain the low incidence of some cancers, including multiple myeloma (8) in developing countries where infection incidences with a large array of microorganisms, including viruses, bacteria and parasites are higher. This hypothesis has also been supported by experimental mouse models, showing a preventive or protective effect of virus and parasites on cancer early development (9–11). However, the mechanisms responsible for such a prevention of cancer development might be quite different from those triggered by infections and leading to a prevention of allergic and autoimmune diseases, that have been proposed to be linked with regulatory immune responses (26).

To analyze mechanisms induced by various infectious agents, it is easier to focus on common early interactions with the immune system rather than to develop many individual models of infections. One of the best studied early interactions of microorganisms with the immune system is their recognition by innate receptors and especially by TLRs. Indeed, those receptors trigger immune responses after recognition of a very large range of bacteria, viruses, yeast and parasites. TLR ligation has been shown to induce divergent effects on the development of various tumors [reviewed in Korneev (27)]. A therapeutic effect of CpG stimulation on mesothelioma growth has been reported previously in a model of mesothelioma using orthotopic xenografts in immunodeficient mice (28). CpG stimulation has also therapeutic effect on pancreas adenocarcinoma and colorectal cancer models (29). TLR9 agonists have been tested in early human clinical trials, including in combined therapies (30). However, TLR9 may also be involved in early promotion of some cancers such as gastric cancer, through enhancement of inflammation and of cell proliferation (31). B cell activity depends on the activation of TLR9 and TLR9 ligands may promote the growth and survival of multiple myeloma cells (32). So far, TLR ligand effects were reported after initiation of cancers. However, to the best of our knowledge, the modulation of the immune microenvironment after TLR ligation, leading to enhanced cancer immunosurveillance prior to the occurrence of any cancer cells, has not been deeply explored. We showed here that ligation of some TLRs, and especially of TLR9, may prevent the development of subsequently inoculated tumor cells in normal immunocompetent animals. CpG stimulation was shown to display preventive effect against plasmacytoma and mesothelioma development, which had not yet been reported. This observation suggests that TLR ligation may be one of the mechanisms by which infections enhance cancer immunosurveillance even before any occurrence of tumor cells. Interestingly, TLR9, in addition to viral DNA, recognizes hemozoin that is produced during infection by *Plasmodium* parasites (33) and these parasites trigger enhanced prevention of early plasmacytoma growth in mice (manuscript in preparation).

Cytotoxic lymphoid cells include NK cells, innate lymphocytes such as NKT cells and cytolytic T-cells (CTLs). While NK and NKT cells are fully innate, in the sense that they can recognize various targets without finely specific stimulation by a unique antigen, interestingly CTLs can also be stimulated non-specifically by cytokines (14) and therefore be part of a more general innate response. NK and NKT cells have been reported to play a major role in cancer immunosurveillance (12, 13) and have therapeutic activity in patients with multiple myeloma (34). They may also infiltrate mesothelioma and kill mesothelioma cells (35). Both NK cells, NKT cells and a subpopulation of CD8+ T-cells that share the capacity of early non-cognate response, including IFN-γ production express ASGM1 (36). Therefore, mice treatment with depleting anti-ASGM1 antibody provides information on the role of these cell populations. Their preventive role against early cancer development has been reported in mouse models of myeloma and mesothelioma after infection (9, 11) and was confirmed here after TLR9 ligation. It would be interesting to discriminate the respective role of these cell populations in future studies.

The mechanisms by which TLR ligation leads to innate cytotoxic cell activation remain to be determined. Expression of TLR mRNA in NK cells depends on their subset, state of activation and localization, both in mice and humans (37, 38). Although most TLRs have been found to be expressed on NK cells, expression of TLR1 seems at the highest levels, followed by TLR2, 3, 5, 6, while expression of TLR4 and 7 is very low (38). It has been reported that vaccinia virus infection directly activates NK cells through TLR2 signaling in the presence of accessory cytokines (39). However, NK cell activation after TLR ligation does not necessarily require TLR expression on NK cells, since it may be triggered indirectly by other cell populations that express these innate receptors (37). This might be the case for dendritic cells, able to secrete IL-12 after CpG stimulation (40). Similarly, anti-tumoral activity of NKT cells is enhanced after activation of TLR9, probably through a mechanism that involves dendritic cells (41). Moreover, activated NKT cells express TLR3, 5, 7, and 9 and can be directly stimulated by their ligands (42). TLR3, 7 and 9 are also expressed on CD8+ T-cells where their expression can be modulated by infection (43). An indirect effect on B-1 B cells that can promote tumor cell killing (44) cannot be excluded.

Although IL-12 alone had little effect on a multiple myeloma mouse model, it could enhance the efficiency of additional therapy (45). By using an antibody recognizing the complete heterodimeric cytokine, rather than a mere IL-12 p40 subunit, which is shared with IL-23 (23), our results strongly suggest the crucial role of this molecule in the enhancement of cancer immunosurveillance induced by ligation of TLR, and especially of TLR9 prior to the occurrence of cancer cells. It is quite plausible that IL-12 is produced by dendritic cells or macrophages in response to CpG stimulation. However, so far, our attempts to determine the cellular origin of IL-12 after *in vivo* CpG-ODN stimulation have not been successful. As reported by many studies, IL-12 activates functions of innate cytotoxic cells, and especially their IFN-γ production. However, the latter cytokine may be dispensable in the involvement of these cells in cancer immunosurveillance.

In conclusion, our study indicates that TLR, and especially TLR9 ligation might be a mechanism by which infections can enhance a more efficient state of cancer immunosurveillance, even in the absence of tumor cells. This enhanced state of cancer immunosurveillance involves cytotoxic lymphoid cells that can include NK cells, NKT cells, and subpopulations of CD8+ T-cells, activated through innate mechanisms. It requires the production of IL-12, while IFN-γ, also necessary after some TLR stimulations, is dispensable in others. This capacity of some infections to enhance cancer immunosurveillance is balanced by the inverse effect of other infectious agents such as *Schistosoma* parasites that can inhibit cytokine production by NK cells and suppress the prevention of plasmacytoma early growth induced by CpG-ODN treatment (manuscript in preparation). Therefore, the final effect of infections on cancer immunosurveillance will depend on the susceptibility of cancer on the preexisting state of immunosurveillance, and on the type of infections, with their ability to modulate positively or negatively cytotoxic cell activity. These elements, combined with the well-known direct inducing effect of some infections on cancer development should be taken into consideration in epidemiological studies on cancer prevalence in developing versus industrialized countries.

## Data availability statement

The raw data supporting the conclusions of this article will be made available by the authors, without undue reservation.

## Ethics statement

The animal study was reviewed and approved by the Comité d’Ethique facultaire pour l’Expérimentation Animale–Secteur des Sciences de la Santé–Université catholique de Louvain (ref. 2014/UCL/MD/008 and 2018/UCL/MD/007).

## Author contributions

MM: investigation, data analysis, and writing. PS and A-SC: investigation and writing. JV: conceptualization and writing; J-PC: conceptualization, data analysis, and writing. All authors contributed to the article and approved the submitted version.

## References

[1] de Martel C Ferlay J Franceschi S Vignat J Bra F Forman D Global burden of cancers attributable to infections in 2008: a review and synthetic analysis. *Lancet Oncol.* (2012) 13:607–15. 10.1016/S1470-2045(12)70137-7 22575588

[2] Oikonomopoulou K Brinc D Kyriacou K Diamandis EP. Infection and cancer: revaluation of the hygiene hypothesis. *Clin Cancer Res.* (2013) 19:2834–41. 10.1158/1078-0432.CCR-12-3661 23536438

[3] Abel U Becker N Angerer R Frentzel-Beyme R Kaufmann M Schlag P Common infections in the history of cancer patients and controls. *J Cancer Res Clin Oncol.* (1991) 117:339–44. 2066354 10.1007/BF01630717 PMC12200960

[4] Albonico HU Bräker HU Hüsler J. Febrile infectious childhood diseases in the history of cancer patients and matched controls. *Med Hypotheses.* (1998) 51:315–20. 10.1016/s0306-9877(98)90055-x 9824838

[5] Kölmel KF Gefeller O Haferkamp B. Febrile infections and malignant melanoma: results of a case-control study. *Melanoma Res.* (1992) 2:207–11. 1450674 10.1097/00008390-199209000-00009

[6] Kölmel KF Pfahlberg A Mastrangelo G Niin M Botev IN Seebacher C Infections and melanoma risk: results of a multicentre EORTC case-control study. *Melanoma Res.* (1999) 9:511–9. 10596918

[7] Krone B Kölmel KF Grange JM Mastrangelo G Henz BM Botev IN Impact of vaccinations and infectious diseases on the risk of melanoma–evaluation of an EORTC case-control study. *Eur J Cancer.* (2003) 39:2372–8. 10.1016/s0959-8049(03)00625-7 14556930

[8] Becker N. Epidemiology of multiple myeloma. In: Moehler T Goldschmidt H editors. *Multiple Myeloma. Recent Results in Cancer Research.* Vol. 183. Berlin: Springer (2011). p. 25–35. 10.1007/978-3-540-85772-3_2 21509679

[9] Thirion G Saxena A Hulhoven X Markine-Goriaynoff D Van Snick J Coutelier J-P. Modulation of the host microenvironment by a common non-oncolytic mouse virus leads to inhibition of plasmacytoma development through NK cell activation. *J Gen Virol.* (2014) 95:504–9. 10.1099/vir.0.063990-0 24739273

[10] De Beule N Menu E Bertrand MJM Favreau M De Bruyne E Maes K Experimental African trypanosome infection suppresses the development of multiple myeloma in mice by inducing intrinsic apoptosis of malignant plasma cells. *Oncotarget.* (2017) 8:52016–25. 28881710 10.18632/oncotarget.18152 PMC5581009

[11] Mandour M Soe PP Uyttenhove C Van Snick J Marbaix E Coutelier J-P. Lactate dehydrogenase-elevating virus enhances natural killer cell-mediated immunosurveillance of mouse mesothelioma development. *Infect Agents Cancer.* (2020) 15:30. 10.1186/s13027-020-00288-6 32391074 PMC7203855

[12] Dunn GP Koebel CM Schreiber RD. Interferons, immunity and cancer immunoediting. *Nat Rev Immunol.* (2006) 6:836–48. 17063185 10.1038/nri1961

[13] Iannello A Thompson TW Ardolino M Marcus A Raulet DH. Immunosurveillance and immunotherapy of tumors by innate immune cells. *Curr Opin Immunol.* (2016) 38:52–8. 26686774 10.1016/j.coi.2015.11.001 PMC4715905

[14] Berg RE Crossley E Murray S Forman J. Memory CD8+ T cells provide innate immune protection agains *Listeria monocytogenes* in the absence of cognate antigen. *J Exp Med.* (2003) 198:1583–93. 10.1084/jem.20031051 14623912 PMC1592647

[15] Akira S Takeda K. Toll-like receptor signalling. *Nat Rev Immunol.* (2004) 4:499–511. 15229469 10.1038/nri1391

[16] Paget C Mallevaey T Speak AO Torres D Fontaine J Sheehan KCF Activation of invariant NKT cells by Toll-like receptor 9-stimulated dendritic cells requires Type I interferons and charged glycosphingolipids. *Immunity.* (2007) 27:597–609. 10.1016/j.immuni.2007.08.017 17950005

[17] Cancro M Potter M. The requirement of an adherent cell substratum for the growth of developing plasmacytoma cells in vivo. *J Exp Med.* (1976) 144:1554–67. 10.1084/jem.144.6.1554 1003103 PMC2190485

[18] Davis MR Manning L Whitaker D Garlepp MJ Robinson BW. Establishment of a murine model of malignant mesothelioma. *Int J Cancer.* (1992) 52:881–6. 1459729 10.1002/ijc.2910520609

[19] Markine-Goriaynoff D Hulhoven X Cambiaso CL Monteyne P Briet T Gonzalez M-D Natural killer cell activation after infection with lactate dehydrogenase-elevating virus. *J Gen Virol.* (2002) 83:2709–16. 12388806 10.1099/0022-1317-83-11-2709

[20] Billiau A Heremans H Vandekerckhove F Dillen C. Anti-interferon-gamma antibody protects mice against the generalized Shwartzman reaction. *Eur J Immunol.* (1987) 17:1851–4. 10.1002/eji.1830171228 3121360

[21] Thirion G Coutelier J-P. Production of protective gamma-interferon by natural killer cells during early mouse hepatitis virus infection. *J Gen Virol.* (2009) 90:442–7. 19141454 10.1099/vir.0.005876-0

[22] Jones LL Chaturvedi V Uyttenhove C Van Snick J Vignali DA. Distinct subunit pairing criteria within the heterodimeric IL-12 cytokine family. *Mol Immunol.* (2012) 51:234–44. 10.1016/j.molimm.2012.03.025 22487722 PMC3341524

[23] Gaignage M Uyttenhove C Jones LL Bourdeaux C Chéou P Mandour MF Novel antibodies that selectively block mouse IL-12 enable the re-evaluation of the role of IL-12 in immune protection and pathology. *Eur J Immunol.* (2021) 51:1482–93. 10.1002/eji.202048936 33788263

[24] Klinman DM Yi AK Beaucage SL Conover J Krieg AM. CpG motifs present in bacteria DNA rapidly induce lymphocytes to secrete interleukin 6, interleukin 12 and interferon gamma. *Proc Natl Acad Sci U S A.* (1996) 93:2879–83. 10.1073/pnas.93.7.2879 8610135 PMC39727

[25] Rook GAW. Review series on helminths, immune modulation and the hygiene hypothesis: the broader implication of the hygiene hypothesis. *Immunology.* (2009) 126:3–11. 10.1111/j.1365-2567.2008.03007.x 19120493 PMC2632706

[26] Rook GAW. Hygiene hypothesis and autoimmune diseases. *Clin Rev Allergy Immunol.* (2012) 42:5–15. 22090147 10.1007/s12016-011-8285-8

[27] Korneev KV. TLR-signaling and proinflammatory cytokines as drivers of tumorigenesis. *Cytokine.* (2017) 89:127–35. 10.1016/j.cyto.2016.01.021 26854213

[28] De Cesare M Sfondrini L Pennati M De Marco C Motta V Tagliabue E CpG-oligodeoxynucleotides exert remarkable antitumor activity against diffuse malignant peritoneal mesothelioma orthotopic xenografts. *J Transl Med.* (2016) 14:25. 10.1186/s12967-016-0781-4 26810896 PMC4727408

[29] Okada H Takahashi K Yaku H Kobiyama K Iwaisako K Zhao X In situ vaccination using unique TLR9 ligand K3-SPG induces long-lasting systemic immune response and Synergizes with systemic and local immunotherapy. *Sci Rep.* (2022) 12:2132. 10.1038/s41598-022-05702-0 35136110 PMC8825851

[30] Zhangchi D Jian L Yuzhang W. Toll-like receptor-9 agonists and combination therapies: strategies to modulate the tumour immune microenvironment for systemic anti-tumour immunity. *Br J Cancer.* (2022) 127:1584–94. 10.1038/s41416-022-01876-6 35902641 PMC9333350

[31] Tang K McLeod L Livis T West AC Dawson R Yu L Toll-like receptor 9 promotes initiation of gastric tumorigenesis by augmenting inflammation and cellular proliferation. *Cell Mol Gastroenterol Hepatol.* (2022) 14:567–86. 10.1016/j.jcmgh.2022.06.002 35716851 PMC9307956

[32] Xu Y Zhao Y Huang H Chen G Wu X Wang Y Expression and function of Toll-like receptors in multiple myeloma patients: toll-like receptor ligands promote multiple myeloma cell growth and survival via activation of nuclear factor κB. *Br J Haematol.* (2010) 150:543–53. 10.1111/j.1365-2141.2010.08284.x 20629663

[33] Coban C Ishii KJ Kawai T Hemmi H Sato S Uematsu S Toll-like receptor 9 mediates innate immune activation by the malaria pigment hemozoin. *J Exp Med.* (2005) 201:19–25. 15630134 10.1084/jem.20041836 PMC2212757

[34] Rossi F Fredericks N Snowden A Allegrezza MJ Moreno-Nieves UY. Next generation natural killer cells for cancer immunotherapy. *Front Immunol.* (2022) 13:886429. 10.3389/fimmu.2022.886429 35720306 PMC9202478

[35] Sottile R Tannazi M Johansson MH Cristiani CM Calabro L Ventura V NK- and T-cell subsets in malignant mesothelioma patients: baseline pattern and changes in the context of anti-CTLA-4 therapy. *Intern J Cancer.* (2019) 145:2238–48. 10.1002/ijc.32363 31018250

[36] Kosaka A Wakita D Matsubara N Togashi Y Nishimura S-I Kitamura K AsialoGM1+CD8+ central memory-type T cells in unimmunized mice as novel immunomodulatory of IFN-γ-dependent type 1 immunity. *Intern Immunol.* (2007) 19:249–56. 10.1093/intimm/dxl140 17229818

[37] Adib-Conquy M Scott-Algara D Cavaillon J-M Souza-Fonseca-Guimaraes F. TLR-mediated activation of NK cells and their role in bacterial/viral immune responses in mammals. *Immunol Cell Biol.* (2014) 92:256–62. 10.1038/icb.2013.99 24366517

[38] Noh JY Yoon SR Kim T-D Choi I Jung H. Toll-like receptors in natural killer cells and their application for immunotherapy. *J Immunol Res.* (2020) 2020:2045860. 10.1155/2020/2045860 PMC7199539 32377528

[39] Martinez J Huang XP Yang YP. Direct TLR2 signaling is critical for NK cell activation and function in response to vaccinia viral infection. *PLoS Pathog.* (2010) 6:e1000811. 10.1371/journal.ppat.1000811 20300608 PMC2837413

[40] Pompei L Jang S Zamlynny B Ravikumar S McBride A Hickman SP Disparity in IL-12 release in dendritic cells and macrophages in response to Mycobacterium tuberculosis is due to use of distinct TLRs. *J Immunol.* (2007) 178:5192–9. 10.4049/jimmunol.178.8.5192 17404302

[41] Prasit KK Ferrer-Font L Burn OK Anderson RJ Compton BJ Schmidt AJ Intratumoural administration of a NKT cell agonist with CpG promotes NKT cell infiltration associated with an enhanced antitumour response and abscopal effect. *OncoImmunology.* (2022) 11:2081009. 10.1080/2162402X.2022.2081009 35712122 PMC9196710

[42] Villanueva AI Mansour Haeryfar SM Mallard BA Kulkarni RR Sharif S. Functions of invariant NK T cells are modulated by TLR ligands and IFNα. *Innate Immun.* (2015) 21:275–88. 10.1177/1753425914527327 24934453

[43] Hammond T Lee S Watson MW Flexman JP Cheng W Fernandez S Toll-like receptor (TLR) expression on CD4+ and CD8+ T-cells in patients chronically infected with hepatitis C virus. *Cell Immunol.* (2010) 264:150–5. 10.1016/j.cellimm.2010.06.001 20579979

[44] Haro MA Dyevoich AM Phipps JP Haas KM. Activation of B-1 cells promotes tumor cell killing in the peritoneal cavity. *Cancer Res.* (2019) 79:159–70. 10.1158/0008-5472.CAN-18-0981 30224373 PMC6318009

[45] Wang X Feng X Wang J Shao N Ji C Ma D Bortezomib and IL-12 produce synergetic anti-multiple myeloma effects with reduced toxicity to natural killer cells. *Anticancer Drugs.* (2014) 25:282–8. 10.1097/CAD.0000000000000058 24300915

